# The impact of a formal complaint on Dutch dentists’ professional practice: a survey study

**DOI:** 10.1186/s12903-016-0295-8

**Published:** 2016-09-29

**Authors:** Josef J. M. Bruers, Brigitte A. F. M. van Dam, Ronald C. Gorter, Michiel A. J. Eijkman

**Affiliations:** 1 Department of Social Dentistry and Behavioural Sciences, Academic Centre for Dentistry Amsterdam (ACTA), University of Amsterdam and VU University, Gustav Mahlerlaan 3004, 1081 LA Amsterdam, The Netherlands; 2 Department of Research and Information, Royal Dutch Dental Association (KNMT), Nieuwegein, The Netherlands

**Keywords:** Formal complaint, Patient dentist communication, General dental practice, Dentist

## Abstract

**Background:**

A complaint from a patient can have a serious impact on the well-being of dentists. Little is known, however, about the nature and the extent of this impact.

**Methods:**

Therefore in 2013 an anonymous survey was conducted among 955 dentists and dental specialists who were involved in a complaints procedure dealt with by the Complaints Committee of the Royal Dutch Dental Association (KNMT) in the period of mid-2008 to mid-2013.

**Results:**

In total 413 (43 %) of these dentists participated in the study. As a result of a formal complaint 71 % of the respondents experienced a considerable impact in their professional practising, while 52 % stated that it had (also) seriously influenced their attitude towards colleagues and patients. Furthermore, 60 % (also) mentioned effects of a complaints procedure regarding their mental and/or physical well-being.

**Conclusions:**

Being confronted with a formal complaint from a patient leads to a considerable impact on dentists’ professional practice and personal well-being. It is remarkable this did not only pertain to a ‘negative’ impact, but also to a ‘positive’ impact. Despite unpleasant feelings, several dentists regarded the complaint as a ‘wake-up call’. Furthermore, given the relatively high number of successful mediation attempts it can be concluded that this form of complaint handling appears to be a successful way of solving problems that have arisen between patients and dentists.

## Background

This article is an edited translation of a previously published original article written in Dutch [1]. In 2012 nearly 8 out of 10 (77 %) Dutch citizens consulted a dentist, which they did on average 2.5 times [2]. By and large this amounts to well over 32.5 million contacts between patients and dentists per year. In general, these contacts go well and are satisfactory to the patient [3]. Nevertheless, it does happen that feelings of discomfort, annoyance, or anger about the care rendered arise in a patient. This may be the result of mistakes that may have been made, missed appointments, conduct experienced as a rebuff, too high expectations of the treatment, inadequate organisation of care, uncertainty about who performs the treatment, or a difference of opinion on the size of the bill [4]. Boers & Sanaan investigated 401 complaints dealt with by the Central Complaints Committee (CKC) within the complaints procedure of the Royal Dutch Dental Association (KNMT) in the period of 2005-2011 [5]. They categorised 826 complaint components, on which the CKC had given a verdict. It appeared that 49 % were related to dental technical and 50 % to organisational aspects or patient rights.

The complaints procedure of the KNMT meets the requirements such as laid down by the Dutch *Health Sector Client Complaints Act* and is meant for members of the KNMT, the largest professional organisation of dentists in the Netherlands with about 7,200 registered individual dentists and dental specialists [6, 7]. This amounts to 83 % of the total population of 9,258 dentists and dental specialists, aged 64 or younger with a home and/or work address in the Netherlands [8]. In addition, the second largest professional organisation, the Association of Dutch Dentists (ANT) also has a complaints procedure complying with the law. The ANT has a membership of approximately 2,300 registered individual dentists and dental specialists and their Dental Complaints Committee deals with patient complaints for 1,500 of their registered members [9].

An overview of the total number of complaints against Dutch dentists filed annually is not available. Besides the KNMT and ANT complaints procedures there is also the possibility to lodge a complaint with the regional health care disciplinary boards. Furthermore, an as yet unknown number of complaints are lodged against dentists in the civil courts and with the Public Health Care Inspectorate. In addition, when determining the number of complaints against dentists, the fact that more and more institutions have their own boards of complaint should be taken into consideration. However, in 2013 the KNMT complaints procedure dealt with 957 letters of complaint, 454 of which were in fact followed up in a procedure as a formal complaint [10]. In that year with the ANT this concerned 223 complaints, 111 of which were found viable with 50 resulting in a formal procedure [11]. In 2006 it was estimated that on average Dutch dentists are confronted with a complaint resulting in a procedure once in their professional career [12]. Based on the before mentioned figures it can be assumed that the average number of complaints has increased to several times in a dentists’ career.

From some personal experiences it becomes clear that dentists are not often aware of the possibility that they could become involved in a complaint procedure and that, if this does happen, it may have a serious impact. Sleepless nights, thoughts going round in circles and health problems are mentioned, as well as the time a procedure takes and the stressful build-up to the hearing. Feelings of insecurity taking away the pleasure of work and the tendency to see every patient as a potential threat is also reported [13]. In this dentists are not unique: similar experiences of other Dutch care providers have been recorded [14]. Furthermore, several international studies also report that receiving a medical complaint has a significant negative impact on a doctor, ranging from stress, significant risks of moderate or severe depression and adjustment disorder to also drug and alcohol abuse, anxiety and other forms of physical illness [15–19].

While there is some data available on the nature and seriousness of the impact of a complaint on care providers, little is actually known when it concerns dentists. More knowledge is however desirable: it may contribute to raising awareness within the profession that one may be faced with a formal complaint and should be prepared how to best cope with this, both personally and professionally. In addition, more knowledge can be used to inform dentists - and dental students - about how complaints can best be prevented. Eventually this will contribute to a higher quality of patient care. Given the sparse knowledge on this topic, the aim of the present study was investigate the impact of a formal complaint (and the following procedure) on dentists’ professional practising and personal well-being.

## Methods

The study was built up in two phases. It started in the spring of 2012 with a pilot study, in which semi-structured oral interviews were held with 17 dentists and dental specialists who had been in a KNMT complaints procedure [13, 20]. The outcome of this pilot served as input for a quantitative investigation by a questionnaire among a larger group of dentists who faced a complaint which was dealt with via the KNMT complaints procedure. In view of the sensitivity of the topic it was decided to conduct this part of the study by means of an entirely anonymous survey in writing. The questionnaire was constructed based on the categories that emerged from the pilot study. The categories were on: (a) the nature of the complaint and the course of the procedure; (b) the consequences experienced in various aspects of the dentists’ professional practising after the complaints (procedure); (c) the impact one’s their mental and physical well-being; and (d) how the complaints procedure had been experienced. Because of the anonymous nature of the investigation, some general personal and practice characteristics were asked for. After the concept questionnaire was constructed, it was commented upon by an independent Research Committee of the KNMT, which led to some minor modifications.

The research group was defined by all Dutch dentists and dental specialists against whom a formal complaint had been lodged and dealt with via the KNMT complaints procedure in the period of 2008 up to and including the spring of 2013. For this occasion, exclusive permission from the KNMT Executive Board was granted to address this group. In this period 1 or more complaints against 1,013 dentists and dental specialists were dealt with via successful mediation by one of the Regional Mediation Boards (RBR) and/or via the Central Complaints Committee (CKC). The complaints procedure of the Royal Dutch Dental Association (KNMT) offers patients two options. The first option is mediation between the patient and the dentist by a Regional Mediation Board (RBR) or the Specialist Mediation Board (SBR). In case the mediation failed or the patient refuses to try mediation, the second option is for the patient to file a formal complaint with the Central Complaints Committee (CKC). The CKC can declare the complaint either entirely or partially legitimate or unfounded.

By the end of May 2013 this group was first contacted in writing. They received a letter in which the survey was announced and its background explained. Because the topic might trigger memories people were not overly keen to be questioned about, dentists who would rather not participate were offered the opportunity to report this: 58 prospective participants in the survey did so. The remaining 955 dentists and dental specialists received the questionnaire a few weeks later. Ultimately, after two reminders 413 (43 %) of them responded.

As for gender, age group and place of qualification the divisions within the group of respondents did not show significant differences from those in the intended research group. Comparison between respondents and non-respondents with regard to the way in which the complaint had been dealt with and, if applicable, the verdict from the Central Complaints Committee (CKC), also showed no significant differences.

It may safely be assumed that, as far as the aforementioned characteristics of the dentists and dental specialists in the survey are concerned, these constitute a reasonably representative reflection of the entire group of dentists and dental specialists against whom in the research period a complaint was dealt with via the KNMT complaints procedure. The data collected was analysed by means of SPSS [21].

## Results

### Nature and handling of the complaint

In the opinion of the dentists questioned, in 58 % of all cases the cause of the complaint had to do with dissatisfaction with treatment patient, while 29 % refers to patient behaviour (‘an attempt by the patient to ‘squeeze every last drop out of the situation’), 20 % to patient communication, 19 % to costs of treatment and 12 % to another aspect (Table 1).

**Table 1:** Aspects related to the complaint lodged, according to dentists^a^

Regarding treatment		58 %
- No or insufficient result after treatment	24 %	
- Incorrect execution of (parts of) treatment	17 %	
- A complication after treatment, pain during treatment	15 %	
- Critical remarks from a another dentist about treatment rendered	14 %	
- No or insufficient aftercare	4 %	
- Wrong diagnosis	4 %	
- Discontentment about treatment delegated to the oral hygienist	2 %	
Regarding treatment		29 %
- An attempt by the patient to squeeze every last drop out of the situation	29 %	
Regarding treatment		20 %
- Incorrect attitude towards the patient	9 %	
- Insufficient or lacking information about treatment or treatment plan	8 %	
- Bad communication/rapport, incomprehension	5 %	
Regarding treatment		19 %
- Cost of treatment, bills, uncertainty about payments and/or reimbursements	19 %	
Regarding treatment		12 %

*n* = 411

^a^More than one response allowed

More than half (51 %) of the dentists questioned said that the complaint against them had been dealt with via successful mediation by the Regional or Specialists Mediation Boards (RBR/SBR), whereas 17 % stated that the patient had withdrawn the complaint during the procedure. In all other cases (32 %) the complaint had been dealt with by the CKC, directly or after a failed attempt at mediation.

The complaints against 130 dentists who appeared before the CKC were declared entirely legitimate in 23 % of the cases, and partially legitimate in 18 %. 55 % of the cases were declared unfounded; in 3 % of the cases parties still agreed on a settlement and in 1 % the complaint was declared unsustainable. Looking at the total number of complaints that were mediated, withdrawn and dealt with this comes down to 13 % of (partially) legitimate complaints.

Table 2 shows that the perceived nature of a complaint was related to the handling of a complaint. Especially when a complaint was dealt with by the CKC relatively often (34 % versus 23 %) the complaint was related to multiple aspects (treatment, costs, communication and/or patient behaviour) and in relatively few cases to only costs. Furthermore, the perceived nature of a complaint showed no relation with gender and age of the dentists.

**Table 2:** Aspects related to the complaint lodged, according to dentists, in relation to the way in which the complaint was handled

Aspects related to the complaint lodged regarding:^a^	RBR/SBR or withdrawn	CKC	Total
Treatment	32 %	34 %	32 %
Treatment + costs, communication and/or patient behaviour	23 %	34 %	27 %
Costs	11 %	2 %	8 %
Costs + communication and/or patient behaviour	5 %	2 %	4 %
Communication	11 %	6 %	9 %
Communication + patient behaviour	2 %	3 %	2 %
Patient behaviour (+ some other aspect)	10 %	9 %	10 %
Some other aspect/unknown	6 %	10 %	8 %
n	278	130	408

^a^More than one response allowed

*Chi Square test: *p* = 0.00/Cramer’s V = 0.22

### Experienced consequences of the complaint

Being confronted with a complaint had affected 29 % to a large extent or strongly in their personal professional practising, whereas 42 % reported they were affected somewhat. 29 % stated not to be affected (Table 3). Dentists who were involved in the CKC reported more frequently the effects on their professional practising than colleagues whose complaints had been dealt with via mediation (83 % against 65 %; *p* < 0.05).

**Table 3:** Extent to which the complaint has affected dentists, in relation to the way in which the complaint was handled

	RBR/SBR or withdrawn	CKC	Total
Did the complaint affect your professional practising*			
- Not at all	35 %	17 %	29 %
- Somewhat	44 %	38 %	42 %
- Largely/strongly	21 %	45 %	29 %
Did the complaint affect your attitude/feelings towards patients, colleagues and/or co-workers*			
- Not at all	53 %	37 %	48 %
- Somewhat	37 %	45 %	39 %
- Largely/strongly	10 %	18 %	13 %
Did the complaint affect your mental and/or physical well-being*			
- Not at all	43 %	31 %	40 %
- Somewhat	42 %	43 %	42 %
- Largely/strongly	15 %	26 %	18 %
n	279	130	409

*Chi Square test: *p* < 0.05

Table 4 (A) offers an overview of the way in which the complaint had influenced their personal professional practising. With the exception of the experienced result ‘the complaint was a blemish to my good reputation as a dentist’ there proved to be little difference as to the nature in which the complaint was handled.

**Table 4:** Extent to which being confronted with a complaint has affected somewhat, largely or strongly (A) the personal professional practising of dentists, (B) the attitude and/or feeling of dentists towards patients, colleagues and/or staff and (C) the mental and/or physical well-being of dentists, related to the way in which the complaint was handled

	RBR/SBR or with-drawn	CKC	Total
A personal professional practising^a^			
_p_The complaint was a signal to improve/do things differently (‘wake-up call’)	41 %	37 %	39 %
_n_The complaint was a blemish on my good reputation as a dentist*	29 %	44 %	35 %
_n_I started frequent ‘checking and double checking’ during treatment	19 %	22 %	20 %
_n_To me the complaint felt like a personal let down	17 %	17 %	17 %
_n_I became unsure in my professional functioning	16 %	19 %	17 %
_n_I became unsure when rendering certain (types of) treatment	9 %	15 %	12 %
_n_I try to avoid/no longer do certain treatment	9 %	14 %	11 %
_n_I felt indignation, anger, injustice^b^	11 %	8 %	10 %
_n_I am more distrustful, cautious, insecure, selective towards patients^b^	5 %	6 %	5 %
-other	13 %	9 %	11 %
n	181	109	290
B attitude and/or feeling of dentists towards patients, colleagues and/or staff)^a^			
_n_Started seeing every (new) patient as a possible risk*	38 %	55 %	44 %
_p_The complaint has taught me to recognize dissatisfaction of patients earlier	34 %	22 %	29 %
_n_Became more reserved when dealing with (certain similar) patients	26 %	32 %	28 %
_n_Left dealing with certain patients if possible to colleagues or associates	9 %	9 %	9 %
_p_Tried to recognize risky patients better (contact, treatment)^b^	5 %	5 %	5 %
_n_Had less patience in contacts with patients	4 %	4 %	4 %
_n_Felt insecure in my professional functioning towards colleagues or associates	8 %	8 %	8 %
_n_Felt frustrated by disloyal behaviour from colleagues (who caused complaint)^b^	2 %	5 %	3 %
_n_Felt towards my associates that my ‘authority’ as a dentist was undermined	2 %	5 %	3 %
_n_Had less patience in contacts with colleagues or associates	1 %	2 %	1 %
-Other	17 %	16 %	16 %
n	131	82	213
C mental and/or physical well-being^a^			
_n_I had feelings of anger and/or aggression	57 %	47 %	53 %
_n_I felt powerless	44 %	52 %	47 %
_n_I had sleep disorders*	22 %	34 %	26 %
_n_I became suspicious of other people	22 %	18 %	20 %
_n_I felt (continually) scared, tense or tired/stressed*	14 %	27 %	19 %
_n_In the morning I (often) went to work reluctantly	14 %	17 %	15 %
_n_I considered to quit work entirely	10 %	17 %	12 %
_n_I had physical complaints, such as heart palpitations, headaches, sickness	8 %	11 %	9 %
_n_I was afraid of getting a nervous breakdown	5 %	8 %	6 %
- Other	10 %	13 %	11 %
n	158	90	248

_p_Considered as ‘positive’ impact

_n_Considered as ‘negative’ impact

*Difference between dentists, according to the way in which the complaint was handled (RBR/SBR or withdrawn versus CKC), Chi Square test: *p* < 0.05

^a^More than one response allowed

^b^Spontaneous response (expressed in respondents own words)

As is shown in Table 3, more than half (52 %) of the respondents had experienced that the complaint had influenced the attitude and/or the feelings towards other colleagues at work. This was also more applicable to those who ended up with the CKC than to those whose complaint was dealt with through mediation (63 % against 47 %; *p* < 0.05).

From Table 4 (B) it appears that this mainly concerned the attitude and/or the feeling towards patients. Only the feeling of ‘every (new) patient was seen as a possible risk’, which occurred the most, was felt more frequently in dentists who were dealing with the CKC than in those whose complaint had been dealt with through mediation.

Furthermore, 60 % stated that the after effects of the complaint affected their mental and physical well-being (Table 2). Dentists who ended up with the CKC reported this more often than their colleagues whose complaints could be mediated (69 % against 57 %; p < 0.05). Table 4 (C) shows to what kind of mental and physical problems this pertained. Sleeping disorders and feelings of anxiety, stress and fatigue were experienced more by dentists who had been through the ‘CKC trajectory’ than by dentists who had undergone a ‘mediation trajectory’.

All things considered it appears that 21 % of the dentists did not report any impact, 6 % only a positive impact, 42 % a mixed impact and 31 % only a negative impact (Table 5). Moreover dentists whose complaint was dealt with by the CKC reported more often only a negative or a mixed impact than dentists whose complaint had been dealt with through mediation (86 % versus 66 %; *p* < 0.05).

**Table 5:** Impact of a formal complaint as reported by dentists, in relation to the way in which the complaint was handled

Reported impact	RBR/SBR or withdrawn	CKC	Total
Only positive impact	7 %	3 %	6 %
Both positive and negative impact	38 %	49 %	42 %
Only negative impact	28 %	37 %	31 %
No impact reported/impact unknown	26 %	11 %	21 %
n	279	130	409

Chi Square test: *p* = 0.00 / Cramer’s V = 0.21

On the whole, 32 % of the respondents indicated that they were not burdened by unpleasant feelings as a result of the complaint. Others were troubled at the most for some weeks (26 %), some months up to a year (31 %), or longer than a year (11 %). It was noted that only one person was off sick as a result of the complaint, but just for a few days.

### Effects on rendering care

In most of the cases (75 %) the relationship between the dentist and the patient was permanently damaged as a result of the complaint and the complainant had left to go to another dentist; in practically all cases to another practice (Table 6). In 8 % this was not yet clear and also in another 8 % the complainant had left the practice some time ago, the complainant had been treated after referral, or elsewhere during a weekend, or the dentist himself had left and was already working somewhere else. In 9 % of the cases the complainant had remained with the dentist.

**Table 6:** Patient who lodged the complaint did or did not remain with the dentist for treatment, related to the way in which the complaint was handled

	RBR/SBR or withdrawn	CKC	Total
Yes, the patient remained with the dentist for treatment	11 %	3 %	9 %
No, the patient went to another dentist within the practice	1 %	2 %	1 %
No, the patient went to another dentist in another practice	71 %	80 %	74 %
This is not (yet) clear	8 %	9 %	8 %
Not applicable^a^	9 %	6 %	8 %
n	275	129	404

^a^Patient was just passing (weekend, transferred) or left to another dentist (long ago), or the dentist was already working in another practice

Nearly two thirds (64 %) of the dentists said that as a result of the complaint they had become more alert on certain aspects of care or had made changes. Table 7 shows that 53 % of these dentists have become more alert to signals of doubt from patients or too high expectations regarding the treatment. Besides, they are now documenting more accurately what has been discussed with patients about the results and risks of the treatment (49 %). It was also mentioned that they were going to document more treatment data in patient records (46 %), they would pay (even) more attention to patient communication (43 %) and/or better guard their own professional limits when following the patients’ wishes (33 %).

**Table 7:** Aspects of care dentists have changed or have become more aware of as a result of the complaint^a^

- More attentive to patients’ signals of doubt or too high expectations of treatment	53 %
- More accurate documentation of what has been discussed with the patient about the results and risks of treatment	49 %
- More documentation of treatment data in patient records	46 %
- (Even) more attention for patient communication	43 %
- Better safeguarding one’s own professional limits when following patients’ wishes	33 %
- Documentation of patients’ consent with regard to treatment	18 %
- Clearer procedures within the dental team about the recording of patient data	16 %
- Clearer procedures within the dental team about patient communication	15 %
- Earlier consultations with a colleague in case of problems with a patient	8 %
- Earlier consultations with a colleague about treatment or a treatment proposal	6 %
- More careful when expressing opinions to patients about the work of colleagues	3 %
- Other aspect	13 %

*n* = 265

^a^More than one response allowed

## Discussion

In the present study the focus was on the consequences of a complaints procedure for dentists and dental specialists. The questions were not limited to information about the effects on the professional practising of these health care providers and their attitude towards patients, colleagues and/or staff, but also pertained to their mental and/or physical well-being. Eight out of 10 respondents indicated that they had experienced a considerable impact in at least one of these areas. It is remarkable this did not only pertain to a ‘negative’ impact, but also to a ‘positive’ impact. It is true that two thirds of the respondents mentioned unpleasant feelings as a result of the complaint, but at the same time 4 out of 10 dentists (also) regarded the complaint as a ‘wake-up call’. So, the complaints procedure obviously also generates a ‘spontaneous’ effect on the profession with regard to the quality of care [7].

Several studies indicate that in relatively many cases complainants are not satisfied with the outcome of a formal complaint procedure [22]. This has to do with the circumstance that doctors often fail to respond in line with what complainants are looking for: in particular an explanation and/or an apology [23, 24]. The chances of getting an explanation and/or an apology are greater in a more informal handling of a complaint, which therefore seems beneficial to patients. Anyway, this study proves that the more informal ‘mediation trajectory’ leads to less dentists who experience a negative impact. Presumed that also more patients are positive about the outcome when the complaint was handled by mediation, the results of this survey suggest that a two-stage complaints procedure as described meets the needs of patients to some extent and enables them to hold dentists accountable when they are of the opinion that they have not been dealt with adequately [25]. Especially the relatively high number of successful mediation attempts indicates that this form of complaint handling appears to be a successful way of solving problems that have arisen between patients and dentists. In this way more burdensome procedures are being prevented, both for dentists and patients.

Thus from the many successful mediation results it can be derived that in dentist - patient communication there is room for improvement. About half of the respondents indicated they had learned to better improve at this point. This was also suggested by Boers and Sanaan and, for that matter, the respondents themselves also agreed [5]. Several respondents said that as a result of the complaint they are more alert to signals of doubt from patients or too high expectations of treatment, pay more attention to communication with patients, better safeguard their own professional limits when following patients’ wishes and/or make procedures clearer within the dental team about patient communication. In a survey aimed at the way in which dentists deal with rules and regulations regarding patient communication it was noted earlier that there was room for improvement [26]. In connection to the above about half of the dentists questioned also said that in the future they will pay more attention to good documentation of what has been discussed and agreed upon with patients. Both careful communication and adequate documentation should be considered as major tools to help prevent a dentist from being confronted with a formal complaint procedure. It is strongly recommended to emphasize this in both graduate and postgraduate education.

Nobody looks forward to receiving a complaint and therefore it is not strange that unpleasant feelings arise when this happens. Many a dentist does not seem to be upset for too long and sees a complaint as a warning. But it also becomes clear that other dentists will still be seriously ‘out of sorts’ when they are confronted with a complaint. They feel their good reputation is blemished, experience the complaint as a personal let down, lose their self confidence in contacts with patients, colleagues and staff and/or become mentally unbalanced (stress, suspicious, insomnia). It is difficult to describe this in general, since personal characteristics naturally play a part. But that the impact is substantial in a number of cases has perhaps something to do with the fact that in oral health care it often concerns long treatment relationships in which trust has developed. This is in the interest of the patient, but also of the dentist. Silverman et al. point out that ’the development of a long term relationship with patients is experienced by most care providers as the most satisfying aspect of their work’ [27]. A complaint is synonym to a violation to this. Therefore, it can lead to the strong impact dentists are mentioning, certainly when a complaint is lodged unexpectedly and when the patient goes to another dentist, which is often the case. This is also in accordance with the results of research on burnout among dentists which shows that a breach of trust with patients leads to more stress [28]. Here we must indicate that in this survey a relatively `dentist-friendly procedure' has been under investigation, without public hearings and serious measures. It may be assumed that the impact of a disciplinary hearing, which can have a considerably stronger and more formal character, will even be greater. All the more, since this survey already showed that the impact of a ‘heavier’ CKC trajectory had been greater than the impact of a ‘lighter’ mediation trajectory. All in all, the impact Dutch dentists experience when confronted with a formal complaint was in most cases unpleasant, but temporal and in any case less severe than the situation a physician gets after a serious adverse patient event. The impact of such an event is defined by the concept of ‘second victim’, e.g. a health care provider involved in an unanticipated adverse event, a medical error and/or a patient related injury who becomes victimized in the sense that he or she is seriously traumatized by the event [29]. The prevalence of second victims after an adverse event varies from 10 % up to 43 %. Common reactions can be emotional, cognitive, and behavioral [30]. None of the dentists in this survey has mentioned impact that could characterize him or her as a second victim. Generally, adverse events in oral health care are rare, not life-threatening or having serious consequences [31]. Besides, complaints about serious patient events in dentistry are brought to the Disciplinary board and seldom dealt with via the KNMT complaints procedure.

When it concerns patient complaints of course it always goes that prevention is better than the cure. Here, first and foremost, the responsibility lies with the dentist himself. The aspects of care in this survey that dentists have changed as a result of a complaint lodged against them can form a leitmotif for prevention. In short: consideration for and attentiveness towards patients, clear and thoughtful explanation of oral health and the pros and cons of treatment, ‘informed consent’ and adequate documentation can prevent complaints. The Institute of Medicine does not describe in vain good communications with patients as 1 in 6 criteria of good medical care [32]. In this way wishes and preferences of patients can be taken into account and decisions can be made by mutual agreement. In addition clear work procedures within the team and consultations with colleagues in case of troublesome and/or difficult treatment are also important points of attention in the prevention of complaints.

Here contributions can also be made at a more collective level. Of course this already starts in education. Van der Ven et al. advocate that dentists have to be imparted with sufficient knowledge on health care legislation and patient rights in their academic studies and later on via post academic education [7]. In addition, the professional oral health care organisations can also contribute, for example by providing their members with tools for good patient communications (information, training courses) and adequate documentation. All sorts of guidelines and other knowledge documents can help oral health care providers in rendering well-considered care to patients. Good patient information from the professional organisation, by means of a website or a telephone service, can also prevent complaints. That these initiatives emanate a preventive effect is already known from experiences with TIP, a telephone service in the Netherlands where patients can pose all kinds of questions about the treatment (plans) of their dentist. Thus, in 2012 the TIP answered calls from approximately 4,600 people. 58 % of these calls ended in the patient declaring that he/she intended to (re) open the discussion with the dentist, 17 % wanted consultations with a third party and 18 % refrained from further actions. The remaining 6 % (still) intended to lodge a complaint against their dentist [33].

In the perception of several dentists a complaint has to do with a patient who aims to squeeze every last drop out of a situation. From the data of this study it can’t be determined whether this perception is correct. Probably this interpretation stems from the fact that in the Netherlands dental treatment is not or only partially reimbursed for adults. In case of a complaint regarding treatment it is conceivable that dentists experience that patients exploit the situation. Especially, because dentists tend to fail to fully explain the content and costs of a treatment.

This study has some weaknesses, which have to do with the type of data. First of all it concerns retrospective data, representing the recollection of dentists regarding thought, feelings and experiences they had in the past. For some the past could be a few years ago and for others some months ago. Respondents can under- or overestimate their earlier reactions. In any case, this forms a risk with regard to the reliability of the data.

Furthermore, because of the anonymous character of the data collection it is difficult to determine exactly to what extent these results are representative for all the dentists who were confronted with a complaint in the past few years. Besides, it must be kept in mind that patients can also lodge their complaints elsewhere, including e.g. with the regional health care disciplinary boards. In 2013 a total of 104 complaints against dentists were lodged with these disciplinary boards, 27 (26 %) of which were ultimately declared legitimate [34]. It is known, however, that these may include complaints that were also lodged with the KNMT or other complaints procedures. A straightforward comparison between these complaints procedures is therefore not possible. Finally the cross sectional design has its limitations. The study deals with the impact on dentists of a complaint. To fully understand this phenomenon a more comprehensive study in time is required.

Nevertheless, this study yields relevant and new information on the impact of complaints of patients on dentists. This information can be cons id ered acceptably representative since it covers the situation in the Netherlands nationwide over a period of several years. Moreover, from a comparison of a number of relevant characteristics it has been made plausible that the participants in this survey constitute a reasonable reflection of the total population of dentists and dental specialists who were confronted with a complaint through the KNMT complaints procedure.

## Conclusions

Being confronted with a formal complaint from a patient has a serious impact on many dentists. This not only affects the different aspects of professional practise, but also mental and physical well-being. The handling of a complaint through mediation appears to be preferable to dealing with the complaint by means of a verdict from the CKC. Anyway, dentists for whom the complaint was successfully mediated reported fewer ‘negative’ consequences in certain respects. Moreover, it is not just a matter of ‘negative’ impact (stress, blemished reputations, anger etc.), a complaint is often also read as a signal to do things better. To a majority of dentists who are confronted with a complaint this is therefore the reason to change certain matters in the care they render. In doing so, a great deal of attention is paid to improve on communication with patients and on documentation of the care rendered. It is recommended to make this lesson learned also part of dental (post)graduate curricula.

## Abbreviations

ACTA: Academic Centre for Dentistry Amsterdam
ANT: Association of Dutch Dentists
CKC: Central Complaints Committee
KNMT: Royal Dutch Dental Association
RBR: Regional mediation board
SBR: Specialists mediation board
TIP: Dental information point
